# The Influence of Treatment of Inflammatory Arthritis During Pregnancy on the Long-Term Children’s Outcome

**DOI:** 10.3389/fphar.2021.626258

**Published:** 2021-03-18

**Authors:** Cecilia Nalli, Jessica Galli, Daniele Lini, Angela Merlini, Silvia Piantoni, Maria Grazia Lazzaroni, Victoria Bitsadze, Jamilya Khizroeva, Sonia Zatti, Laura Andreoli, Elisa Fazzi, Franco Franceschini, Alexander Makatsariya, Yehuda Shoenfeld, Angela Tincani

**Affiliations:** ^1^ Rheumatology and Clinical Immunology Unit, ASST Spedali Civili and University of Brescia, Brescia, Italy; ^2^ Child Neurology and Psychiatry Unit, Department of Clinical and Experimental Sciences, ASST Spedali Civili and University of Brescia, Brescia, Italy; ^3^ Department of Clinical and Experimental Sciences, University of Brescia, Brescia, Italy; ^4^ Department of Obstetrics and Gynecology, I.M. Sechenov First Moscow State Medical University of the Ministry of Health of the Russian Federation (Sechenov University), Moscow, Russia; ^5^ Obstetric and Gynecology Unit, ASST Spedali Civili of Brescia, Brescia, Italy; ^6^ Department of Medicine ‘B’, The Zabludowicz Center for Autoimmune Diseases, Sheba Medical Center, Ramat Gan, Israel; ^7^ Sackler Faculty of Medicine, Tel-Aviv University, Tel Aviv-Yafo, Israel; ^8^ I.M. Sechenov First Moscow State Medical University of the Ministry of Health of the Russian Federation (Sechenov University), Moscow, Russia

**Keywords:** rheumatoid arthritis, spondyloarthritis, psoriatic arthritis, pregnancy, immunosuppressants, children’s development

## Abstract

The management of reproductive issues in women with inflammatory arthritis has greatly changed over decades. In the 1980–1990s, women with refractory forms of arthritis were either not able to get pregnant or did choose not to get pregnant because of their disabling disease. Hence, the traditional belief that pregnancy can induce a remission of arthritis. The availability of biologic agents has allowed a good control of aggressive forms of arthritis. The main topic of discussion during preconception counselling is the use of drugs during pregnancy and breastfeeding. Physicians are now supported by international recommendations released by the European League Against Rheumatism and the American College of Rheumatology, but still they must face with cultural reluctance in accepting that a pregnant woman can take medications. Patient-physician communication should be centered on the message that active maternal disease during pregnancy is detrimental to fetal health. Keeping maternal disease under control with drugs which are not harmful to the fetus is the best way to ensure the best possible outcome for both the mother and the baby. However, there might be concerns about the influence of the *in utero* exposure to medications on the newborn’s health conditions. Particularly, studies suggesting an increased risk of autism-spectrum-disorders in children born to women with rheumatoid arthritis has raised questions about neuropsychological impairment in the offspring of women with chronic arthritis. As a multidisciplinary group of rheumatologists and child neuropsychiatrists, we conducted a study on 16 women with chronic forms of arthritis whose diagnosis was determined before pregnancy and their 18 school-age children. The children underwent a complete neurological examination and validated tests/questionnaires. Behavioral aspects of somatization and anxiety/depression (internalizing problem) or an “adult profile” were found in nearly one third of children. Children at a high risk of neurodevelopmental problems were born to mothers with a longer history of arthritis and were breastfeed for less than 6 months of age or were not breastfeed at all. No association was found with other maternal characteristics such as autoantibody existence and disease activity during and after the pregnancy.

## Introduction

In the past decades, young women affected by chronic inflammatory arthritis were discouraged of a pregnancy due to the joint pains, fatigue, disability, skeletal deformity and the loss of self-esteem. As a matter of fact, these women have a smaller family size as compared not only with the general population but also with patients with other rheumatic diseases (Østensen, 2004). Nowadays, the general health status of patients with inflammatory arthritis has greatly improved due to the introduction, nearly 20 years ago, of new treatments that are able to switch off inflammation and consequently to control the symptoms and joint damage. Despite this improvement in management, the approach to reproductive issues is an unmet need as a reduced family size has been still observed in men and women with rheumatic diseases (Østensen, 2017b). Several different reasons underlie this phenomenon: disease activity, impaired sexual function, personal choices, and an increased rate of pregnancy loss, even if this problem is not as frequent in patients with inflammatory arthritis as in other rheumatic diseases.

An additional important issue is related to the need of treatment during pregnancy. In an ideal situation, women with inflammatory arthritis should become pregnant when the disease is in a stable remission thanks to the administration of an effective therapy. Treatment should be ideally continued throughout the pregnancy to ensure good outcomes. In fact, in contrast to what was described before the 1990s, we now are aware that pregnancy “*per se*” is not able to induce remission in all the patients with inflammatory arthritis (Østensen et al., 1983).

According to a recent metanalysis published in 2019, nearly 40% of the patients experience a remission state during pregnancy (Atta et al., 2016; Jethwa et al., 2019). Therefore, the majority of the women with inflammatory arthritis can have flares during pregnancy and need to be treated even if pregnancy was initiated in a remission phase. Obviously, this rule will be more stringent when pregnancy took place in a period of active disease, even if this is a relatively rare event because of the above-mentioned reasons. Pregnant women with inflammatory arthritis have an increased need for treatment today than in the past. This situation is related to the fact that pregnancy generally occurs along with patient’s well-being that in the past was achieved only in relatively mild situations responding to traditional drugs. Nowadays, also patients with an aggressive arthritis can reach a well-being state and start a pregnancy if adequately treated with effective biological drugs (Giles et al., 2019).

In these conditions, the known decrease in Th1 activity will not be enough to keep the disease in remission (Østensen et al., 2005).

On the other hand, the puerperium has always been known as a period with a high risk of flares for patients with inflammatory arthritis (Jethwa et al., 2019). This can be particularly frustrating since the mothers need to feel good to look after the newborn. The feeling of being inadequate in taking care of the newborn can facilitate the development of postpartum depression. Therefore, disease control must be achieved with drugs that are compatible with breastfeeding (Ince-Askan et al., 2019a).

The hardest task for the physician is to convince pregnant and nursing patients that taking “safe” drugs is a way to take care of the baby’s wellbeing. If the mother feels well, then the baby will be OK too. Reassuring data about the health and growth conditions of children antenatally exposed to anti-rheumatic drugs are useful for counselling (Bortoluzzi et al., 2021).

This article aims at reviewing the literature and report our experience on the long-term follow-up of children born to patients with inflammatory arthritis exposed to anti-rheumatic drugs before and during pregnancy and breastfeeding.

## The Use of Anti-Rheumatic Drugs During Pregnancy

The control of disease activity is fundamental for a successful pregnancy. In fact, high disease activity is associated with an increased risk of pregnancy complications and adverse outcomes, including preeclampsia, preterm delivery, low birth weight, small for gestational age, and fetal loss (Østensen, 2017a). According to the published data, even if no randomized clinical trials are available in the field of pregnancy, an effective treatment of chronic arthritis is possible with an acceptable safety profile during pregnancy and lactation. The dissemination of this evidence among health professionals and patients is important in order to improve the management of pregnant and lactating mothers with rheumatic diseases.

In 2016, the European League Against Rheumatism (EULAR), and in 2020, the American College of Rheumatology (ACR), published recommendations for a safe use of anti-rheumatic drugs before and during pregnancy and lactation (Götestam Skorpen et al., 2016; Sammaritano et al., 2020). The same was done by the British Society of Rheumatology (Flint et al., 2016a; Flint et al., 2016b).

To date, the available data from the literature and from registries showed that a great number of drugs can be taken by pregnant and lactating women without causing harm to the children in terms of congenital malformations and miscarriages. Glucocorticoids, sulfasalazine, hydroxychloroquine, chloroquine, azathioprine, colchicine, cyclosporine, tacrolimus and intravenous immunoglobulins (IvIg) were reported to be compatible with pregnancy and lactation.

The data about non-steroidal anti-inflammatory drugs in pregnancy are conflicting but, in general, non-selective COX inhibitors are preferable.

Methotrexate, mycophenolate mofetil and cyclophosphamide must be discontinued before conception due to their proven teratogenicity.

Even though biologics have shown efficacy in keeping the disease under control, their safety profile in pregnant women is still under investigation. A recent meta-analysis on this topic found no association between the use of biologics during pregnancy and the risk of congenital malformation, preterm deliveries or serious infections requiring hospitalization in infants’ first year of life, compared with disease-matched unexposed pregnant women (Tsao et al., 2020). The use of adalimumab (ADA), certolizumab pegol (CTZ), infliximab (IFX), and etanercept (ETA) during pregnancy is now widely accepted if clearly needed, based on the results of the pregnancy registries (Ghalandari et al., 2020). All the TNFα inhibitors showed a good safety profile if used in the first half of pregnancy. In fact, even if they differ in their molecular structure, they are all of big molecular size proteins which cannot passively diffuse and reach the fetus during the first trimester of gestation. Based on this assumption, unintended pregnancies exposed to these drugs should not raise any concern. Moreover, it is currently suggested to maintain the treatment during pregnancy, as their discontinuation at a positive pregnancy index has been associated with maternal disease flares during pregnancy (and poorer pregnancy outcomes as a consequence) (Van den Brandt et al., 2017).

Considering their molecular structure which influences their placental transfer, IFX, ADA and ETA may preferentially be discontinued in the second/third trimester. In fact, active transplacental transport of immunoglobulin G (IgG) from mother to infant is mediated by the neonatal receptor for the crystallizable (FcRn), a process that takes place mainly during the second and third trimesters of pregnancy. Differently from the others, CTZ cannot be transferred across the placenta, because it lacks IgG Fc region. For this reason, it has a safer profile during pregnancy with specific studies showing minimal to no transfer into neonatal blood and breastmilk (Clowse et al., 2018; Mariette et al., 2018). Evidence for safety is still scarce for leflunomide, tofacitinib as well as golimumab, abatacept, tocilizumab, ustekinumab, rituximab (RTX), anakinra and canakinumab. Even if no alerts were published about their use before conception or in the first trimester, the general advice is to discontinue their use before a planned pregnancy (Götestam Skorpen et al., 2016; Sammaritano et al., 2020), but the progressive increase of available data could change this attitude, as it has happened for the TNFα inhibitors.

Regarding the use of rituximab in selected severe cases of arthritis, the recommendation is to continue the treatment until conception/early during the first trimester but also during pregnancy in the presence of severe maternal disease. There are no data suggesting teratogenic effects for exposure to RTX during the first trimester, while second/third-trimester exposure is associated with neonatal B cell depletion (Flint et al., 2016a).

The high rate of pregnancies without known outcomes and the high number of lost to follow-up limit the potentiality of global clinical safety databases (Sinclair et al., 2014). The data available for azathioprine, cyclosporine and dexamethasone do not indicate immunosuppression in the exposed children; on the contrary, the administration of biologics after gestational week 30 might increase the risk of postnatal infection (Giles et al., 2019). However, the children exposed to biologics before the week 22 can receive vaccinations according with the standard protocols (Furer et al., 2020). Pharmacological properties of drugs act as a guide for decision to allow breastfeeding, considering the insufficient and heterogeneous documentation regarding clinical practice (Flint et al., 2016a; Götestam Skorpen et al., 2016).

## Lights and Shadows About the Prescription of Anti-Rheumatic Drugs to Pregnant Women With Inflammatory Arthritis

Women suffering from severe arthritis during pregnancy and who were offered to take drugs to relieve their pains often answer, “I will stay in bed for 9 months and bear all the pain of the world, but I do not want to cause any harm to my baby by taking drugs!.” This widespread attitude is driven by the rooted belief that any drug taken during gestation can be harmful to the child. It is no easy task for the physicians to convince the patient otherwise; much has still to be achieved in the physician-patient communication about the use of drugs during pregnancy.

The patients’ knowledge about the compatibility of drugs during pregnancy is extremely limited. According to a recent national survey performed in Italy (Andreoli et al., 2019), even patients with previous successful pregnancies occurring after the disease onset had discordant opinions about this topic. In fact, several of them were not aware of the safety of drugs commonly offered to treat arthritis during pregnancy. As an example, about 30% of the patients did not know that hydroxychloroquine can be taken during pregnancy and lactation without any harm to the child. These findings underline the need of young women with inflammatory arthritis of an exhaustive counselling focusing on reproductive issues but, in particular, on the consequence of treating or not treating their disease (Chew et al., 2019).

Physicians must empower their skills in performing counselling. It is first necessary to clarify to the patients that active arthritis can interfere with fetal growth and cause preterm delivery and small for gestational age infants together with other less frequent pregnancy complications (Andreoli et al., 2019). In contrast with the traditional belief that pregnancy related immunomodulation was able to induce disease remission at least in patients affected by RA, several recent reports underlined that this is true only for a part of the patients, while flares during gestation are recorded in about 29% of patients with RA and 25% of patients with axial spondyloarthropathy (Zbinden et al., 2018). A significantly increased risk of flares is linked to the presence of active disease at conception and to the withdrawal of effective treatments at the beginning of pregnancy. According to these findings, the refusal to take drugs during pregnancy will not favor the baby’s health, but will rather deteriorate the mother’s joints (with the consequent difficulties in neonatal caregiving) and will increase the risk for adverse pregnancy outcomes that can negatively affect the baby’s health. The second aspect is related to the safety of the drugs to be administered in pregnancy. As detailed in the previous paragraph, many drugs routinely employed in Rheumatology are compatible with pregnancy while very few need to be withdrawn before conception because of their possible teratogenicity (Ahmed et al., 2020).

To make the issue easily understandable to patients, it is possible to classify anti-rheumatic drugs used to treat inflammatory arthritis into three categories: 1) drugs that can be continued during pregnancy, 2) drugs that need to be withdrawn, and 3) drugs with no evidence of harm but without sufficient experience for confirming their safety (Table 1). The drugs recently introduced in clinical practice belong to the third group and their administration in pregnant women need to be carefully evaluated by considering the risk/benefit balance in the individual case.

**TABLE 1:** Classification of drug administration to pregnant patients with inflammatory arthritis and their restrictions during pregnancy [adapted from Østensen (2017a)].

Drugs to be stopped before pregnancy	Drugs with insufficient data	Drugs that can be used in pregnancy
Methotrexate (stop at least 3 months before a planned pregnancy), Cyclophosphamide (stop at least 3 months before a planned pregnancy), Mycophenolate Mofetil (stop at least 6 weeks before a planned pregnancy), Warfarin/acenocumarol (discontinuation at positive pregnancy test).	Leflunomide, Mepacrine, Biologic agents other than anti-TNF alfa inhibitors (e.g., rituximab, anakinra, tocilizumab, abatacept, belimumab, ustekinumab), JAK inhibitors, Selective COX-2 inhibitors.	Steroids (oral, intra-articular and intravenous pulses^a^), Hydroxychloroquine, Chloroquine, NSAIDs (until 32 weeks of gestation), Azathioprine, Cyclosporine, Tacrolimus, Sulfasalazine, Colchicine, Intravenous immunoglobulins^a^, anti-TNF alfa inhibitors.

Abbreviations: anti TNF, anti-tumor necrosis factor; NSAIDs, nonsteroidal anti-inflammatory drug.

^a^ For severe organ involvement.

Counselling should include also a discussion about the post-partum period. Patients with RA or spondyloarthropathy must be aware of the high risk of flares (Jethwa et al., 2019) during this delicate period in which they are expected to be able to take care of the newborn. About half of the women affected by RA reported a worsening of joint pain after delivery (Eudy et al., 2018). To explain the high rate of flares observed in the weeks after delivery, some authors focused on the possible effect of high levels of prolactin (Borba et al., 2019) and discussed situations in which breastfeeding should be discouraged (Vieira Borba and Shoenfeld, 2019). However, the recently published ACR guidelines (Sammaritano et al., 2020) underline the benefits of breastfeeding for both the baby and the mother. Therefore, the guidelines recommend encouraging breastfeeding by achieving a good disease control with compatible medications, highlighting the need for more scientific evidence in this specific area. In this respect, a very recent publication underlines the benefit of breast feeding on the neonate immune system: the exposure of children to maternal cells even for a short time was shown to enhance the maturation of neonate T regulatory cells (Wood et al., 2020).

## The Outcome of Children Born to Mothers With Inflammatory Arthritis and Antenatally Exposed to Anti-Rheumatic Drugs

### Outcomes Related to Disease

The information on the children born to mother affected by rheumatic diseases are mainly accumulated by national and international registries. A recent report focuses on the RAPPORT registry, a retrospective anonymous RedCAP survey of peripartum period in females with RA/Psoriatic Arthritis (PsA). Authors collected data on 234 pregnancies (103 patients), 164 pregnancies before and 70 pregnancies after the disease onset. The results included: 96% live births, 1.9% stillbirths, 23% miscarriages and 15% therapeutic abortions. A third of patients had fewer children than desired due to disease activity and medications. Notably, no statistically significant differences occurred between pregnancies before or after RA/PsA diagnosis regarding pregnancy planning, fertility treatment, pregnancy and delivery complications, birth defect frequency or neonatal complications (Dissanayake et al., 2020).

Larger studies, mainly from US registries, showed an increased rate of some complications such as preterm delivery and IUGR that appeared to be related to the active disease during pregnancy. The pathogenic mechanism that allows increased disease activity to cause these complications is not certain yet, although inflammation could be a possible explanation (Littlejohn, 2020).

### Malformations

As previously detailed, according to the available international guidelines, treatment with TNF inhibitors can be offered to pregnant patients. Because of their protein nature, these large molecules (mainly IgG derived) cannot reach the embryo in the first trimester, the critical period for organogenesis, while they can actively reach the fetus in the third trimester, with the possible increased risk of neonatal infections. Most of the data about the risk of malformations in patients with inflammatory arthritis come from the registries.

Recently a nationwide registry-based study including all male live births from RA and SLE in Denmark from 1995 to 2016 was published with the aim to assess the occurrence of cryptorchidism and hypospadias (the most frequent malformations in boys) according to the prenatal disease-state of the mothers. They found among 690,240 boys, 1,026 born from mother with RA and 352 from mothers with SLE. Compared with unexposed boys, these subgroups of children had a higher risk of cryptorchidism: Authors described an adjusted hazard ratio of 1.72 (95% CI: 1.15; 2.57) in the group of RA-children and 1.46 (95% CI: 0.69; 3.06) in the SLE-group. No conclusion could be reached on the risk of hypospadias, due to the low number of events (Knudsen et al., 2020a).

Another recent retrospective cohort study displays similar results: congenital anomalies, small for gestational age and preterm birth were more common in neonates of women with RA compared with controls (Aljary et al., 2020). Unfortunately, data about medication employed both before and during the pregnancy (type, dosage, duration of use) were not available.

### Newborns Infections

One of the main concerns about biological treatment during pregnancy, considering the mechanism of action, is the possible increased risk of infections in newborns. Follow-up studies of anti-TNFα therapy during pregnancy and the risk of infections in the offspring were firstly conducted in women with IBD. IFX and ADA have been detected in the infants’ circulation and may remain detectable up to 6–12 months of life (Julsgaard et al., 2016), while CTZ was not found in neonates (Mariette et al., 2018) and in maternal milk (Clowse et al., 2017).

A recent multicenter study with a mean follow-up of 4 years reported no increase in hospital admission for infections in children born to women with IBD who had been treated at any time during pregnancy or during the 3 months before conception with anti-TNFα compared with women with IBD without anti-TNFα therapy (Chaparro et al., 2018). Similar results were described in a French study that reported no increased risk of pediatric infections in a large cohort of children born to women with IBD treated with anti-TNFα during pregnancy (Luu et al., 2018).

A Canadian study by Vinet et al. evaluated 2,989 children born to women with RA exposed to anti-TNFα during pregnancy, including exposure in the third trimester: the Authors could not detect a significant increased risk for serious infections (Vinet et al., 2018).

On the other hand, Juulsgaard et al. reported that the risk of infection was significantly increased in a cohort of 80 women with IBD treated with ADA or IFX (Julsgaard et al., 2016).

Recently a three-country population-based cohort (Denmark, Sweden, and Finland) was published. Aim of the work was to described the risks of hospital admissions for infection in a cohort of children born to women treated with anti-TNFα during pregnancy compared to women with the same diseases, but not treated during pregnancy, and healthy controls. Data were recorded from the national medical birth registries, patients’ registries and prescribed drug registries. 1,617,886 children were included: 1,027 were born to treated women and 9,346 to women with non-biologic systemic treatment. The results showed an increased rate of hospital admissions due to infections in the first year of life, as well as increased rates for antibiotic prescriptions during the second year of life. There was no differences among different anti-TNFα, and there was no distinct pattern for type of infection (Bröms et al., 2020).

### Vaccinations

Finally, we report some data regarding the response to vaccinations of children and its safety. There are some controversial data to the safety of live vaccines administered in early childhood to the children of mothers exposed to anti-TNFα during pregnancy, but the data are non-conclusive. European and North American guidelines for patients with IBD recommend postponing the use of live vaccines until 6 months post-delivery (Nguyen et al., 2016).

There is at least one reported fatality case due to disseminated tuberculosis infection in an infant who received a Bacillus Calmette‐Guerin for tuberculosis (BCG) vaccination at 3 months of age and whose mother had been taking IFX 10 mg/kg every 8 weeks for Crohn’s disease during all the pregnancy (Cheent et al., 2010; Heller et al., 2011). Recently a French nationwide population-based cohort on the safety of vaccinations during the first year of life in exposed children was published (Luu et al., 2019). The Authors studied 670 children exposed to anti-TNFα during pregnancy of women with IBD: among these exposed children, 88 (13%) received BCG live vaccine before 6 months of life and no adverse events were reported. To note, for the great part of the mothers (61.5%) the time for last exposure to anti-TNFα therapy was ≥26 weeks of gestation.

A recent study in children born to mothers with IBD and exposed to anti‐TNFα *in utero* (IFX and ADA) showed adequate immune serologic response for Hepatitis B vaccination compared to children not exposed. The median anti-TNFα stop week was 25 (IQR 22–29) in the IFX group and gestational week 23 (IQR 22–24) in the ADA group. There are no differences between the groups also in birth outcome (de Lima et al., 2018).

All these data came from IBD mothers that mainly received anti-TNFα therapy during all the course of pregnancy, but we can assume that data could be analogous in women with inflammatory arthritis who usually receive therapy until the 32–34th week of gestation.

## The Long-Term Outcome of Children Born to Women With Inflammatory Arthritis

The long-term outcome of children represents a remarkably interesting area, certainly challenging the investigators.

### Epigenetic

One of the hypotheses is that some adverse event occurring in pregnancy could cause long-term consequences. Pathological alteration of pregnancy could also act “*via*” epigenetic changes in children themselves possibly are involved in long-term consequences (Jones et al., 2019). In this respect, DNA methylation profile of children born to RA mothers was recently investigated. This is a well-studied fetal epigenetic modification, that, in this case, was demonstrated to be related with several risk factors (maternal disease, malnutrition, smoking, placental insufficiency, corticosteroids, folate depletion and cytokines). Samples from 80 children were analyzed (mean age 6.4 years old) from RA mothers and 354 controls. They found some differences between the DNA methylation in the two groups, and, notably, some of the differentially methylated sequences or their nearby genes were associated with cardiovascular or metabolic disease. It can be speculated that DNA methylation could influence the long-term outcome of these children and open new interesting scenarios. It remains unknown whether the identified associations are causal, and if so, whether they are caused by either the disease or the treatment during pregnancy (Ince-Askan et al., 2019b).

### Maternal Condition in Puerperium

Maternal disease state after delivery could also influence the long-term outcome of the children. Smeele et al. recently investigated women affected by rheumatoid arthritis. In their work, high Health Assessment Questionnaire (HAQ) scores in the first trimester, high disease activity in the first trimester, disease duration and the presence of erosive disease were predictive for developing parenting disability after delivery. Unfortunately, whether mothers’ disabilities could affect the children was not investigated (Smeele et al., 2020).

### Autoimmune Disease, Congenital Heart Defect and Neurodevelopmental Disorders

A 2017 a review focused on the long-term outcome of children born to women affected by RA and Systemic Lupus Erythematosus. As previously reported, these data were also derived by national registries, with all the limitations related to this data collection. The authors concluded that these children could be potentially at increased risk of neurodevelopmental disorders, congenital heart defects and autoimmune diseases, compared with children from the general population. Genetic factors, obstetric complications, maternal autoantibodies, cytokines and drugs might be related to the increased risk. However, the extremely low rate of these events is overall reassuring: the absolute risk is small, and patients should not be discouraged from having children (Vinet and Bernatsky, 2017).

### Autism

A possible relationship between RA in mothers and autism spectrum disorders were hypothesized. In a systematic literature review, 70 articles on this topic were included. A potentially increased risk of developing autism in children born to mothers with RA was described, although data are limited and conclusions not definitive (Wojcik et al., 2017).

### School Achievement

School performance can be considered a good indicator of a possible cognitive impairment and it was investigated in children born to mothers with chronic arthritis. In fact, a recent Danish study examined the overall cognitive development of children born from RA mothers by comparing their school test scores with those of their peers. They linked data from the National School Test Register and national registries of disease and evaluated about 1,000 children. There were no differences between the groups in reading test scores while RA exposed children scored poorer in mathematics tests. Furthermore, there was no appreciable difference in children exposed to maternal seropositive RA or between children exposed to preclinical RA versus children exposed to established RA. To note, no relationship with therapy was described, probably due to missing data (Knudsen et al., 2020b).

## Our Experience: The Neuropsychological Outcome of School-Age Children Born to Women With Inflammatory Arthritis

As far as we know, no studies have described the neuropsychological status of children born to mothers with RA by means of validated questionnaires and direct neurological physical examination.

In our Hospital, a multidisciplinary team (rheumatologists and child neuropsychiatrists) has carried out a study to evaluate the school performance and the psychological profile of a group of children born to mothers with chronic arthritis. The study included 16 women (11 RA, 3 PsA, 2 ankylosing spondylitis - SpA) with the diagnosis before pregnancy and their 18 school-age children (F/M = 1/1, median age 8 years, range 6–14). Information about maternal disease and treatment during pregnancy were collected from medical records (pregnancies had been prospectively followed-up in our Pregnancy Clinic). Three validated questionnaires were administered to all the mothers at the time of the children follow-up: the Child Behavior Check List (CBCL 6–18; Achenbach and Rescorla, 2001), a screening tool, used to identify clinical, borderline and normative behaviors in the children; the State-Trait Anxiety Inventory (STAI; Spielberg et al., 1970) to measure trait and state anxiety of the mothers during all their life and at the time of their child evaluation; and the Edinburgh Postnatal Depression Scale (EPDS; Cox et al., 1987) to identify mothers who may have postpartum depression; in our work this was a retrospective evaluation with possible bias, even if a vivid memory of the post-natal period was reported by all the mothers.

Notably, according to the clinical reports, at the time of the interview, maternal disease was effectively treated and all the patients were found in clinical remission.

A child neuropsychiatrist performed a neurological physical examination of all the children. In addition, the Wechsler Intelligence Scale for Children-IV (Wechsler, 2003) to evaluate cognitive level was administered to all the children; this instrument provides a Full Scale Intelligence Quotient and four Composite or Index Scores (Verbal Comprehension, Perceptual Reasoning, Working Memory and Processing Speed Index). Finally, standardized batteries of tests to evaluate academic learning (Test AC-MT 6-11, 2002; Cornoldi and Cazzola, 2003; Sartori et al., 2007; Cornoldi and Carretti, 2016) were also administered. We evaluated speed and accuracy of text reading (MT-3 test, 56) and, only for children scored greater than the Dyslex-ia spectrum cut-off, the speed and accuracy of words and nonsense words reading (DDE-2 test, items 2 and 3,57) were also evaluated. Words and nonsense words writing (DDE-2, items 6 and 7, 57) were measured as well as the arithmetic abilities (Test AC-MT 6-11, 2002; Cornoldi and Cazzola, 2003). The neurological physical examination and the intelligence quotient were normal in all the children as well as the academic skills.

According to CBCL questionnaire, behavioral aspects of somatization and anxiety/depression (internalizing problem) were found in six children (33%) and an “adult profile” in 5 (28%). We also found that all children were less involved in sports or playing activities than peers but more involved in school activities, as they seemed to invest more energies in intellectual functioning.

We searched for associations between the risk of behavioral difficulties in children and the maternal characteristics (Table 2).

**TABLE 2:** The personality profile of children according to maternal disease characteristics.

Maternal characteristics	Child personality (*n* = 18)		*p* value
	Not at risk of neuro-developmental problems (*n* = 7)	At risk of neuro-developmental problems (*n* = 11)	
Disease duration at conception (median years; IQR)	2 (2; 5)	10 (7.5; 13)	**<0.05**
Active disease during pregnancy (*n*, %)	1 (14%)	6 (55%)	ns
Active disease in the post-partum period (*n*, %)	5 (71%)	8 (73%)	ns
Severe disease activity in the post-partum period (*n*, %)	3 (43%)	8 (73%)	ns
Positive antiphosholipid antibodies (*n*, %)	2 (29%)	2 (18%)	ns
Positive rheumatoid factor (*n*, %)	2 (29%)	3 (27%)	ns
Positive anti-citrullinated peptides (*n*, %)	0 (0%)	1 (9%)	ns
Employment (*n*, %)	7 (100%)	7 (64%)	ns
Positive EPDS questionnaire (depression) (*n*, %)	2 (29%)	5 (45%)	ns
Post-partum depression reported by mothers (*n*, %)	2 (29%)	5 (45%)	ns
Positive STAI questionnaire (anxiety) (*n*, %)	2 (29%)	6 (55%)	ns
Anxiety reported by mothers (*n*, %)	3 (43%)	5 (45%)	ns
Psychological support received during the post-partum period (*n*, %)	1 (14%)	4 (36%)	ns
Difficulty in caregiving, as rated by the patient (*n*, %)	3 (43%)	6 (55%)	ns
Duration of Breastfeeding (months median, IQR)	6 (6, 8.5)	1 (0; 4.5)	<0.05
Breastfeeding for less than 6 months (*n*, %)	1 (14%)	9 (82%)	<0.05
STOP Breastfeeding because of the need of taking drugs (*n*, %)	3 (43%)	5 (45%)	ns

Abbreviations: EPDS, Edinburgh Postnatal Depression Scale; IQR, interquartile range; ns, not significant; STAI, State-Trait Anxiety Inventory.

Our results seem to show that children at high-risk of behavioral problems were born to mothers with a longer history of arthritis and were breastfed for less than 6 months of age or were not breastfed at all (see Table 2). No association was found with other maternal characteristics such as autoantibody presence and disease activity during and after pregnancy.

On the maternal side, according to STAI questionnaire, we identified seven mothers (44%) with a state of anxiety both at the time of the study and during the all life.

The results from EPDS were quite similar reported a significant score for the risk of post-partum depression.

We found significant associations between active disease during pregnancy and difficulties in caregiving and breastfeeding (*p* = 0.005 and *p* = 0.0128, respectively). In addition, only twelve mothers breastfed their children (67%). The patients recollected a diffuse state of anxiety mainly due to their active disease as a major obstacle toward a normal parenting experience.

This study has several limitations: 1) the number of examined children is small; 2) maternal disease data are retrospective and based on clinical records; 3) the psychological evaluation of the mothers was performed only in a single time-point at the time of the interview and a serial evaluation of their psychological status was not available.

However, it has also the strength of an original investigation about the risk of cognitive and behavioral problems of children born to mother with inflammatory arthritis. These data are in line with our previous preliminary study (Bomba et al., 2010) already supporting the hypothesis that the mother’s disease impaired child handling and care-taking in the first years of life interfering with the parenthood and baby holding, impacting on maternal-fetal attachment and on the children development.

Even taking into account the above quoted limitations, our study suggests that children born to women with chronic arthritis are normal from a neurological, cognitive and learning point of view. However, they may display behavioral difficulties, that we found related to a complicated maternal experience during pregnancy and the post-partum period, even if it cannot be excluded the influence of maternal chronic disease long-life (Bomba et al., 2010). Therefore, it could be reasonable to assess closely the psychological status of patients during pregnancy and after delivery, along with the activity of the rheumatic disease. The prompt management of an eventually occurring post-partum depression could be of great help to both the mother and the baby and possibly prevent the behavioral problems observed later on. In addition, if a child displays any difficulty during school years, the mother should be encouraged to refer to specialists and be reassured that the early diagnosis yields the best outcomes for the child.

## Conclusion

In women suffering from inflammatory arthritis, the importance of preconception counselling, of the treatment and of the follow up during pregnancy is today well recognized because active disease can be responsible for the increased risk of preterm delivery and intrauterine growth restriction.

If we exclude methotrexate, conventional anti-rheumatic drugs and TNF-inhibitors used in the treatment of patients with inflammatory arthritis can be prescribed during pregnancy and are not linked to an increased risk of malformations although minor malformations associated to maternal RA have been reported (Götestam Skorpen et al., 2016; Knudsen et al., 2020a; Sammaritano et al., 2020).

The transient immunosuppression possibly occurring in some neonates exposed to new biological drugs, does not seem to be of great clinical relevance. However, according to the current recommendations, live vaccines should be avoided in the first 6 months of life.

Despite these recent achievements, patients are often afraid of taking drugs during pregnancy. Certainly, information about the long-term outcome of children exposed *in utero* can increase their compliance and confidence to the prescribed treatment.

The overall occurrence of autoimmune disease, heart congenital defects and neurodevelopmental problems seem rare, although autism disorders were reported. Children of mothers affected by RA seem to have some difficulties in mathematic tests, but not in reading or writing. These data were derived by the results of school tests and no information on maternal treatment was available. Most importantly, none of these children was directly examined.

The direct neurological examination of a small cohort of school age children from patients affected by inflammatory arthritis allowed us to highlight some minor behavioral alterations related to anxiety/depression often yielding an “adult profile.” The affected children performed better in schoolwork than in social/physical activities, possibly reflecting the maternal limitations in everyday life. The drug regimens during pregnancy were not related to the occurrence of these problems. Rather, the disease activity during pregnancy and puerperium, and the lack of breastfeeding seems to play an important role in the onset of behavioral problems. Therefore, our data can suggest that a safe and effective treatment of maternal disease during pregnancy and puerperium is needed to ensure not only a good pregnancy outcome but also a physiological development of the children.
